# Comparative analysis of Borrelia’s Defence mechanisms and their impact on genetic manipulation of low-passage isolates of *Borrelia afzelii* and *Borrelia garinii*

**DOI:** 10.1016/j.crmicr.2025.100543

**Published:** 2025-12-27

**Authors:** Margarida Ruivo, Anna-Margarita Schötta, Theresa Stelzer, Michael Reiter, Michiel Wijnveld

**Affiliations:** Institute for Hygiene and Applied Immunology, Center for Pathophysiology, Infectiology and Immunology, Medical University of Vienna, Kinderspitalgasse 15 1090 Vienna, Austria

**Keywords:** Restriction-modification system, *Borrelia*, Bacteriophages, Transformation efficiency

## Abstract

*Borrelia,* a highly prevalent tick-borne pathogen, has a genome with a linear chromosome and numerous linear and circular plasmids. There are three groups of *Borrelia*: Lyme borreliosis, relapsing fever, and Echidna-reptile. In Europe, *Borrelia afzelii* and *Borrelia garinii* are the main causative agents of Lyme borreliosis.

The primary defence mechanism of bacteria against bacteriophages and other invading DNA elements is the restriction-modification system (RMS), which discriminates between native and foreign DNA based on their distinct methylation patterns.

This present study compares the RMS of all the *Borrelia* species available in the REBASE database. Additionally, it investigates the effect of the RMS on the transformation efficiency of low-passage *B. afzelii* and *B. garinii* isolates.

Upon comparing the RMS of 18 *Borrelia* species, differences in the number, location and characteristics of genes were observed between groups. Given that Lyme borreliosis species exhibit higher genomic plasticity, we hypothesise that they possess a greater number of RMS genes to ensure functionality of the RMS even if some plasmids are lost.

In this study, we demonstrate a large increase in transformation efficiency of low-passage strains by using an in vitro methylated shuttle vector, confirming our hypothesis that the RMS of *Borrelia* recognises pre-methylated vectors as native DNA.

The knowledge gained in this study contributes to the understanding of *Borrelia* defence mechanisms and provides possible explanations for the relatively low transformation efficiency observed in previous studies. Consequently, in vitro methylation can serve as a valuable tool for facilitating studies involving genetic manipulation of *Borrelia.*

## Highlights

• The *Borrelia* restriction-modification system (RMS) differs by species group.
• The RMS affects the transformation efficiency of *Borrelia.*
• Increase in transformation efficiency observed with an in vitro methylated vector.
• Transformation efficiency increased in low-passage *Borrelia* strains.
• In vitro methylation can be a facilitating tool for *Borrelia* genetic manipulation.

## Graphical abstract

**Figure:**
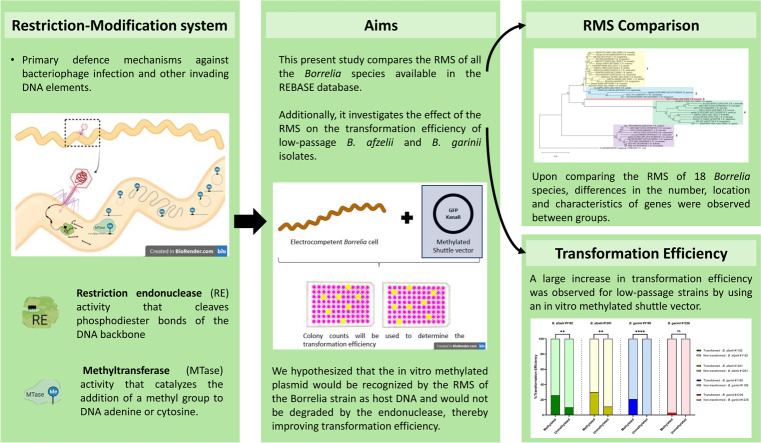

## 1 Introduction

The bacterial Restriction-Modification system (RMS) is a primary defence mechanism against bacteriophage infection and other invading DNA elements, and, thus, could inhibit genetic transformation. This system is present only in unicellular organisms, including bacteria (Vasu et al., 2019). A typical RMS comprises two distinct enzymatic activities: a restriction endonuclease activity that cleaves the phosphodiester bonds of the DNA backbone and a methyltransferase activity that catalyses the addition of a methyl group to DNA adenine or cytosine. Both enzymes have identical or overlapping recognition sequences, protecting the host DNA from endonuclease cleavage by methylation of the recognition site. Conversely, the endonuclease quickly fragments incoming foreign DNA, which does not have the same methylation pattern as the host (James et al., 2016; Ruivo et al., 2024; Vasu et al., 2019).

These RMS systems are classified into four different types: I, II, III, and IV. This classification is based on the subunit structure, oligomeric status, recognition sequence, cleavage characteristics, cofactor requirements, mode of action and use of a modified or unmodified substrate. A type I RMS has three functions: restriction, modification and specification. This system forms two configurations: (i) one with two methyltransferase subunits, two endonuclease subunits, and one specificity subunit and (ii) a second one with two methyltransferase subunits and one specificity subunit. The first configuration can exert both endonuclease and methyltransferase activity, and the second one has only methyltransferase activity. However, for both configurations, a specificity protein is necessary to recognise sequences on both DNA strands. A type II RMS consists of two separate proteins (endonuclease and methyltransferase) that act independently of each other. Usually, a type II endonuclease cleaves DNA at or near its recognition site. A type III RMS is encoded by two genes, *mod* and *res*. This system has two configurations: one consists of two methyltransferase subunits (M_2_), and the second one consists of two methyltransferase subunits combined with one endonuclease subunit (RM_2_). The dimeric methyltransferase (M_2_) and/or the trimeric (RM_2_) hemi-methylate the DNA at an asymmetric recognition site. The trimeric configuration also has an endonuclease function that cleaves the DNA. After cleaving, the enzyme remains bound to the still intact recognition site. Finally, a type IV RMS only encodes for an endonuclease that recognises 5mC-modified DNA with low sequence selectivity, restricting Dcm^+^ phage infection (Vasu et al., 2019).

Whereas endonucleases are a highly diverse group of enzymes, methyltransferases fall into one of three types: adenine methyltransferase (^m6^A), cytosine methyltransferase (^m4^C) and cytosine pyrimidine ring methyltransferase (^m6^C). All three types can occur in type II RMS. However, in type I and III RMS, only ^m6^A has been reported (Madhusoodanan & Rao, 2010).

Our work focuses on *Borrelia,* one of the most prevalent tick-borne pathogens. *Borrelia* is a member of the Spirochaetes phylum whose natural infectious cycle alternates between tick vectors and vertebrate hosts (Kurokawa et al., 2020; Stanek et al., 2012). Depending on the transmitted *Borrelia* species, these spirochetes can cause Lyme borreliosis (LB) or relapsing fever (RF) in humans (Koutantou et al., 2024).

Lyme borreliosis is the most prevalent tick-borne disease in the northern hemisphere, with over 200,000 cases per year in Western Europe (Koutantou et al., 2024; Marques et al., 2021). This disease is caused by a group of related spirochetes, *Borrelia burgdorferi* sensu lato (*Bbsl)*, that are transmitted by specific *Ixodes* spp. ticks (Stanek et al., 2012). *Borrelia afzelii* and *Borrelia garinii* are the most frequent species detected in *Ixodes ricinus* and *Ixodes persulcatus* ticks and are the principal causative agents of LB in Europe, with other species playing a less prominent role. In contrast, *B. burgdorferi* sensu stricto (*Bbss*) is the main causative agent of this disease in the United States of America and Canada (Marques et al., 2021).

Relapsing fever is usually associated with two vectors: lice and ticks. It is mainly caused by specific *Borrelia* species (of the RF group) transmitted to humans by soft ticks, with two exceptions. *Borrelia recurrentis,* which is transmitted from human to human by the body louse, and *Borrelia miyamotoi,* which is transmitted by hard-bodied ixodid tick species (Koutantou et al., 2024; Lopez et al., 2021).

Recently, a third group of *Borrelia* has been described, the Echidna-reptile group. This group is mostly associated with reptile and echidna hosts and with *Amblyomma, Bothriocroton* and *Hyalomma* as tick vectors. So far, this group includes two species, *Borrelia turcica* and *Candidatus* Borrelia tachyglossi, that do not cluster phylogenetically with any of the LB or RF group *Borrelia* species (Gabriele Margos et al., 2018).

*Borrelia* exhibit remarkable genomic features that distinguish them from most bacteria, including the other *Spirochaetes*. The genome consists of a linear chromosome of approximately 900 kb and over 20 linear (lp) and circular (cp) plasmids (Kurokawa et al., 2020; Schwartz et al., 2021). Gene expression is tightly regulated to facilitate the spirochetes’ adaptation to switch between different environments, i.e. the arthropod and vertebrate hosts (Kurokawa et al., 2020). In recent years, some efforts have been made to identify and characterise these genes (Chiappa et al., 2022; Faith et al., 2024; Shan et al., 2021). However, due to the complex genome*,* limitations persist for genetic studies of both LB and RF *Borrelia,* including low frequencies and efficiencies of transformation (Rosa & Jewett, 2020). Currently, most studies have been using high-passage laboratory strains (Chan et al., 2015; Lawrenz et al., 2002). However, LB *Borrelia* tend to lose plasmids during in vitro propagation, resulting in laboratory strains significantly deviating from the strains circulating in nature with regard to their plasmid content (G. Margos et al., 2017). The plasmid content from the RF and Echidna-reptile species also varies from species to species. Nevertheless, these species do not tend to lose plasmids in laboratory conditions, unlike the LB species. This demonstrates that the species from these two groups have a higher plasmid stability, even between strains (Gilmore et al., 2021).

Several studies have shown a direct correlation between the activity of the RMS and transformation efficiency (C. Chen et al., 2025; Finn et al., 2021; Huang et al., 2025; Shon et al., 2025). They hypothesised that the RMS confers a barrier to the introduction of DNA, preventing genetic manipulation (James et al., 2016; Kawabata et al., 2004; Ruivo et al., 2024). We demonstrated that bypassing the RMS defence is necessary to increase transformation efficiency (Ruivo et al., 2024).

In the present work, we identified and compared the RMS systems of different *Borrelia* species, including the LB group, RF group and Echidna-reptile group species and investigated the effect of this system on the transformation efficiency of low-passage *B. afzelii* and *B. garinii* isolates.

## 2 Materials and methods

### Identification of hypothetical restriction-modification system genes and phylogenetic analysis

The RMS genes of the different Borrelia species were predicted using the restriction enzyme database website (http://rebase.neb.com last accessed on 2025.04.30). BLAST analysis (2025.04.30) of the hypothetical RMS genes of each species was performed to compare the genes from all available borrelial genomes. The sequences of all RMS genes were obtained from the National Center for Biotechnology Information (NCBI) website. All sequences were aligned using Muscle alignment. Tree-based phylogenetic analyses were performed using a Maximum likelihood method and the Tamura-Nei (1993) model. A maximum likelihood tree was built from 100 bootstrap replicates of the sequences obtained previously. A total of 19 sequences from the RF group, 2 from the Echidna-reptile group and 22 from the LB group were included. As an outgroup, a type II RMS gene of *Escherichia coli* was used.

### In vitro methylation of pBSV2_OspA_GFP

To investigate the transformation efficiency in low passage isolates, the plasmid pBSV2_OspA_GFP (P. E. Stewart et al., 2001) was methylated as previously described to confer the same methylation pattern as *Borrelia* native DNA (Ruivo et al., 2024). In summary, we started by transforming pBVS2_OspA_GFP into a *dam^−^*/*dcm^−^ E. coli* strain containing a vector encoding for a functional RMS for *Borrelia* (bafPKo_H0010) and plated out on ampicillin and kanamycin-containing LB agar plates (50 µg/mL each).

From the plates grown overnight at 37°C, three colonies were picked and grown overnight in Lysogeny Broth medium containing only kanamycin (50 µg/mL) and incubated at 37°C in a shaking incubator (180 rpm) overnight. The potentially methylated plasmids were extracted using the GeneJet Plasmid Miniprep kit (Thermo Fisher Scientific, Vienna, Austria). The methylation was confirmed by digestion with methylation-sensitive restriction enzymes: DpnI, MboI and Sau3AI (New England Biolabs, Frankfurt, Germany), following the manufacturer’s protocol. DpnI cleaves adenine-methylated GATC sites, MboI cleaves unmethylated GATC sites, and Sau3AI cleaves all GATC sites regardless of adenine methylation but is blocked by methylated cytosine. The resulting DNA fragments were resolved by agarose gel electrophoresis, as done previously (Ruivo et al., 2024).

### Transformation efficiency analysis in low-passage isolates

The borrelial patient isolates were kindly donated by Brian Crowe and TAKEDA Pharmaceuticals (Vienna, Austria). These isolates were cultured in a modified BSK II medium (Reiter et al., 2015).

The methylated pBSV2_OspA_GFP was used to transform *B. afzelii* and *B. garinii* patient isolates. As a control, an unmethylated pBSV2_OspA_GFP was used. Preparation of electrocompetent patient isolate cells and transformation were carried out as described by Samuels 1995 (Samuels, 1995). The cells were resuspended in 1 mL of prewarmed modified BSK II medium after electroporation and subsequently added to a 15 mL tube with 9 mL of the same medium for incubation overnight at 34°C. As a starting concentration, a dilution of 10 spirochetes per well was plated in each well of a 96-well plate with kanamycin (50 µg/mL). As a viability control, the same dilution was plated in a 96-well plate without antibiotics. All plates were incubated at 34°C for 14 days. To determine transformation efficiency for each isolate, wells with spirochete growth were counted. Additional confirmation of positive wells was carried out by visualising the expressed GFP in the green fluorescent spirochetes using a Nikon Eclipse TE300 Inverted Fluorescence Phase Contrast Microscope (Nikon GmbH, Vienna, Austria) using a 40x objective. Transformation efficiency was calculated as a ratio between the number of wells positive in antibiotic-containing plates originating from methylated and unmethylated plasmids, normalised by the viability control.

To evaluate the difference in the transformation efficiency when a methylated plasmid is used in comparison with an unmethylated plasmid, the transformant number was normalized against the viability rate of the control. Chi-square and Fisher´s exact test were then performed on both transformation efficiency values before and after normalization. For this statistical analysis, GraphPad Prism, version 10.6.0 for Windows (GraphPad Software, San Diego, California, USA, www.graphpad.com) was used.

## 3 Results

### Comparison of the RMS genes of different *Borrelia* species

REBASE analysis was performed for a total of 18 available *Borrelia* species within the database, 7 species associated with LB (*B. afzelii, B. bissettii, B. burgdorferi, B. garinii, B. mayonii, B. spielmanii* and *B. valaisiana*), 10 species with RF (*B. coriaceae, B. crocidurae, B. duttonii, B. hermsii, B. hispanica, B. miyamotoi, B. parkeri, B. persica, B. recurrentis,* and *B. turicatae*) and one species of the Echidna-reptile group (*B. turcica).*

The number of predicted RMS genes differed between the three groups, as shown in Table 1. In general, LB species have up to four RMS encoding genes, all of which are located in linear plasmids, except for *B. spielmanii,* whose genome encodes for two RMS genes, one located in the chromosome and one in a linear plasmid. All these genes encode for a type II RMS, which means they encode for a methyltransferase and an endonuclease that work independently of each other. In *Borrelia* of the LB group, a high similarity (93.5 %) was identified in the BLAST analysis on the amino acid level of the genes bafPKo_H0010, bspa14S_H0025, bgaPBr_H0006, bgaPBr_K0029, bmayo_05140, bb_e02 and bbiDN127_H0007, indicating that this RMS gene is conserved among species of this group. Interestingly, *B. garinii* has two genes that are homologous, bgaPBr_K0029 and bgaPBr_H0006. Also, in both *B. mayonii* and *B. bissettii,* two genes were identified in each with a similarity of 96.4 % on the amino acid level. All the other genes only share up to 60 % of similarity.

**Table 1:** RMS genes predicted using REBASE.

Associated Disease	Borrelia species	No of genes	Gene name	Genome location	Type	Accession Number*
Lyme Borreliosis	B. afzelii	4	bafpko h0010	Lp28–3	II	AEL70449.1
			bafpko aa0003	Lp28–7	II	AEL70481.1
			bafpko i0015	Lp28–4	II	AEL70543.1
			bafpko q0015	Lp32–10	II	AEL70543.1
	B. bissettii	4	bbidn127_e0002	Lp25	II	AEL19439.1
			bbidn127_h0007	Lp28–3	II	AEL19468.1
			bbidn127_i0001	Lp28–4	II	AEL19486.1
			bbidn127_ad0001	Lp56	II	AEL19622.1
	B. burgdorferi	4	bb_e02	Lp25	II	ACK74187.1
			bb_h09	Lp28–3	II	AAC66000.1
			bb_k02a	Lp36	II	AAC66031.1
			bb_q67	Lp56	II	AAF07736.2
	B. garinii	4	bgapbr k0029	Lp36	II	ACL34484.1
			bgapbr h0006	Lp28–3	II	ACL34751.1
			bgapbr f0006	Lp28–1	II	ACL34905.1
			bgafar04 f0013	Lp28–1	II	ACL35060.
	B. mayonii	3	bmayo_05140	Lp17	II	A0AAC9KWY3 (No Protein ID available)
			bmayo_05080	Lp25	II	APT00366.1
			bmayo_04970	Lp28–4	II	APT00425.1
	B. spielmanii	2	bspa14s_pa0106	Chromosome	II	EEF83970.1
			bspa14s_h0025	Lp28–3	II	ACN53413.1
	B. valaisiana	3	bvavs116_o0003	Cp32–2–7	II	ACN52728.1
			bvavs116_e0047	Lp25	II	ACN52871.1
			bvavs116_h0117	Lp28–3	II	ACN52993.1
Relapsing Fever	B. coriaceae	3	bcco53_000464	Chromosome	Methyltransferase	UPA16323.1
			bcco53_001526	Lp31	II	UPA17340.1
			bcco53_001613	Lp34	II	UPA17416.1
	B. crocidurae	2	bcd_0463	Chromosome	Methyltransferase	AHH06529.1
			bcd_1611	Unknown	II	AHH07677.1
	B. duttonii	1	bdu_467	Chromosome	Methyltransferase	ACH93408.1
	B. hermsii	2	axx13_02325	Chromosome	Methyltransferase	ANA43271.1
			axx13_v07	Lp47	II	ANA43957.1
	B. hispanica	2	bhicriorfarmp	Unknown	II	AYOU01000037.1 (No Protein ID available)
			bhicriorfbmp	Chromosome	Methyltransferase	
	B. miyamotoi	2	cno12_01935	Chromosome	Methyltransferase	ATQ17518.2
			bmi16orfap	Lp16–12	II	WDE70433.1
	B. parkeri	1	bpa_0099100	Chromosome	Methyltransferase	AHE62785.1
	B. persica	3	bpeno12orfcmp	Chromosome	Methyltransferase	AYOT01000146.1 (No Protein ID available)
			bpeno12orfarmp	Unknown	II	AYOT01000047 (No Protein ID available)
			bpeno12orfermp	Unknown	II	AYOT01000232 (No Protein ID available)
	B. recurrentis	1	bre_470	Chromosome	Methyltransferase	ACH94702.1
	B. turicatae	2	btbtcam1_02335	Chromosome	Methyltransferase	WVN91633.1
			btbtcam1_07245	Lp29–1	II	WVN92560.1
Echidna-reptile group	B. turcica	2	db313_00320	Chromosome	Methyltransferase	AYE35959.1
			db313_05335	Lp32	II	AYE36923.1

⁎ Accession number is the corresponding Protein ID in NCBI.

Relapsing fever *Borrelia* and Echidna-reptile species have two RMS genes, one present in the chromosome and the other in a linear plasmid. While the plasmid encodes for a type II RMS, the chromosome encodes for a methyltransferase only. *Borrelia coriaceae* and *B. persica* deviate from this pattern in that they have three genes, two of which are in plasmids (type II RMS) and one in the chromosome (methyltransferase). *Borrelia duttonii* and *B. recurrentis* have one gene present in the chromosome (methyltransferase).

Regarding the RF *Borrelia*, BLAST analysis on an amino acid level identified a similarity of 94.6 % of all the methyltransferase genes present in the main chromosome. On the other hand, the RMS genes encoded by plasmids only share up to 79.4 % of similarity. Interestingly, the RMS gene present in the linear plasmid of *B. miyamotoi* has a 93.4 % similarity with the conserved RMS gene mentioned previously of LB *Borrelia* strains.

**Fig. 1:**
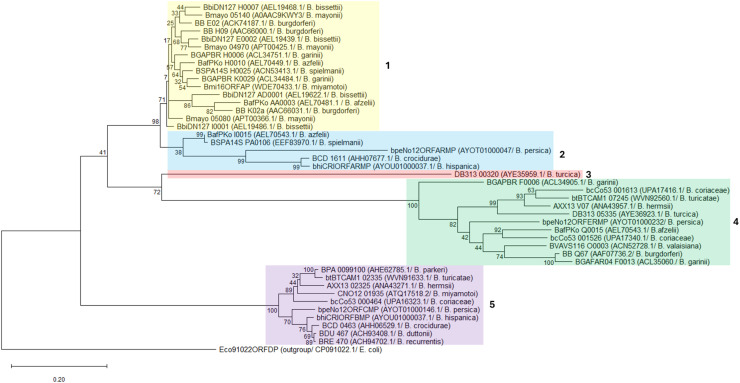
Phylogenetic tree of Borrelia based on the RMS genes. All sequences were aligned using Muscle alignment, and a phylogenetic tree was constructed using the Maximum likelihood method and the Tamura-Nei (1993) model. For the tree, 100 bootstrap replicates of the sequences obtained in REBASE were used. The colours represent the four different clusters observed. As an outgroup, an RMS gene of E. coli was used.

We also constructed a phylogenetic tree to observe the phylogenetic relationship between the RMS genes of the three *Borrelia* groups, resulting in five clades (Fig. 1). The first clade is composed of most of the RMS genes of LB species and one gene of *B. miyamotoi*, all of which are located on linear plasmids. The second clade is composed of three genes present in the plasmids of three RF species (*B. crocidurae, B. hispanica* and *B. persica*) and two genes of LB species (*B. afzelii* and *B. spielmanii*). The third clade is composed only of the RMS gene present in the plasmid of *B. turcica.* The fourth clade is composed of different RMS genes, all of which are present in linear plasmids but belong to different *Borrelia* groups. Finally, the fifth clade clusters all RMS genes present on the chromosome of species of the RF group *Borrelia*.

### Transformation efficiency in patient (low passage) *Borrelia* strains

A pre-methylated, previously described plasmid carrying a green fluorescent protein under the Flab promoter was used to transform early passage patient isolates (P. E. Stewart et al., 2001). We chose four *Borrelia* patient isolates, two *B. afzelii* patient isolates (*B. afzelii* #1192 and *B. afzelii* #1201) and two *B. garinii* patient isolates (*B. garinii* #1195 and *B. garinii* #1226).

These patient isolates were chosen for their difference in the number of RMS genes. *Borrelia afzelii* #1192 has only one gene, and *B. afzelii* #1201 has all four genes. On the other hand, *B. garinii* #1195 has two genes, and *B. garinii* #1226 has three.

**Fig. 2:**
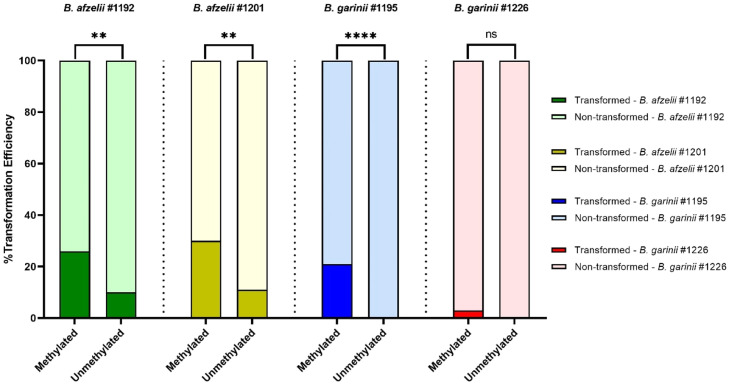
Transformation efficiency of Borrelia strains, normalised with viability control. For B. afzelii #1192 (green), we extrapolated that 30 % (30/100) of the viable spirochetes were successfully transformed when a methylated plasmid was used, compared to 11 % (11/100) for the unmethylated plasmid. For B. afzelii #1201 (yellow), 26 % (26/100) of the viable spirochetes were successfully transformed with a methylated plasmid compared to 10 % (10/100) for the unmethylated plasmid. For B. garinii #1195 (blue), 21 % (12/56) of the viable spirochetes were successfully transformed with a methylated plasmid, compared to 0 % (0/59) for the unmethylated plasmid. For B. garinii #1226, 3 % (3/97) of the viable spirochetes were successfully transformed with methylated plasmid compared with 0 % (0/89) for the unmethylated plasmid. To evaluate the difference in the transformation efficiency, chi-square and Fisher´s exact test were performed (**, ρ<0.01; ****, ρ<0.0001; ns, ρ>0.05). For the statistical analysis and the graph, GraphPad Prism, version 10.6.0 for Windows (GraphPad Software, San Diego, California, USA, www.graphpad.com) was used.

A difference in transformation efficiency was observed in all four *Borrelia* patient isolates when they were transformed with a methylated vector, pBSV2_OspA_GFP, in comparison to an unmethylated vector. In *B. afzelii* #1192, we observed 30.2 % (58/192) of positive transformants when a methylated vector was used and 11 % (22/192) with an unmethylated vector (p<0.001). In *B. afzelii* #1201, we observed 26 % (49/192) of positive transformants with a methylated vector in comparison to 10 % (19/192) with an unmethylated vector (p<0.001). In the viability control of both isolates, we saw a borrelial growth of 100 %, meaning that 192 out of 192 wells exhibited growth. Normalising our transformation efficiency results, taking the viability of each spirochetes strain into account after electroporation transformation (Fig. 2), we extrapolated that for *B. afzelii* #1192, 30 % (30/100) of the viable spirochetes were successfully transformed when a methylated plasmid was used, compared to 11 % (11/100) for the unmethylated plasmid (p<0.01). For *B. afzelii* #1201, 26 % (26/100) of the viable spirochetes were successfully transformed with a methylated plasmid, compared to 10 % (10/100) for the unmethylated plasmid (p<0.01). It shows that methylation improves the transformation efficiency in *B. afzelii* low-passage strains.

The difference in transformation efficiency was more drastic in the *B. garinii* isolates. In *B. garinii* #1195, we observed 12 % (23/192) of positive transformants when a methylated vector was used and no positive wells with an unmethylated vector (p<0.001). Similarly, in *B. garinii* #1226, we observed 3 % (5/192) of positive transformants with a methylated vector and no positive wells with an unmethylated vector (p=0.061). However, in this case, the viability control was not 100 %: *B. garinii* #1195 showed 56 % viable cells (107/192) when the methylated vector was used and 59 % viability (114/192) with an unmethylated vector. For *B. garinii* #1226, it was 97 % (186/192) with the methylated vector and 89 % (170/192) with the unmethylated vector.

**Fig. 3:**
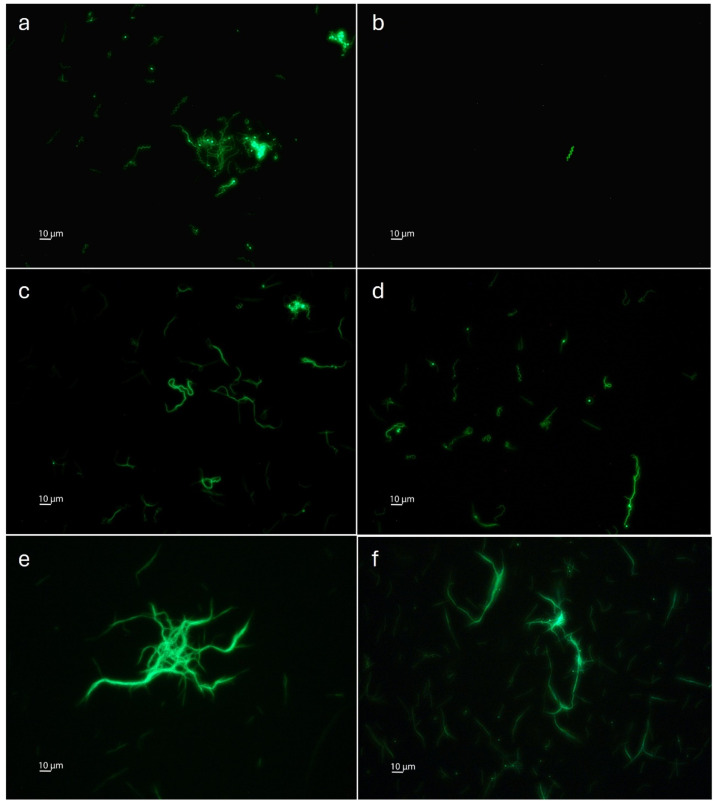
Fluorescence of spirochetes transformed with a GFP plasmid. B. afzelii #1192 transformed with in vitro methylated (a) and with unmethylated pBSV2_OspA_GFP (b). B. afzelii #1201 transformed with in vitro methylated (c) and with unmethylated pBSV2_OspA_GFP (d). B. garinii #1195 transformed with in vitro methylated pBSV2_OspA_GFP (e). B. garinii #1226 transformed with in vitro methylated pBSV2_OspA_GFP (f). All cultures were grown in modified BSK II medium at 34°C. All photos were taken using a Nikon Eclipse TE300 Inverted Fluorescence Phase Contrast Microscope using a 40x objective.

For the *B. garinii* strains, the data show a clear improvement in transformation efficiency (Fig. 2). For *B. garinii* #1195, 21 % (12/56) of the viable spirochetes were successfully transformed with a methylated plasmid, compared to 0 % (0/59) for the unmethylated plasmid (p<0.0001). Finally, for *B. garinii* #1226, 3 % (3/97) of the viable spirochetes were successfully transformed with methylated plasmid compared with 0 % (0/89) for the unmethylated plasmid (p=0.2462). For this strain, the difference between methylated and unmethylated was not statistically significant (p>0.05). Future analysis on this specific strain is necessary to understand the lower transformation efficiency. Successful transformation of all patient isolates was additionally confirmed by visualisation of fluorescent green spirochetes (Fig. 3).

## 4 Discussion

Previous studies identified and characterised the RMS system of *Bbss* (Kawabata et al., 2004; Rego et al., 2011)*, B. afzelii* (Ruivo et al., 2024)*, B. garinii* (Ruivo et al., 2024) and *B. hermsii* (James et al., 2016). For *Bbss,* most studies identified and characterised the two genes, bb_e02 and bb_q67, as RMS genes (Rego et al., 2011). In our REBASE analysis, we predicted two additional genes belonging to this gene family, bb_h09 and bb_k02a. In a study by Rego et al. 2011 (Rego et al., 2011), it was observed that the loss of the plasmids encoding bb_e02 (lp25) and bb_q67 (lp56) genes resulted in a significantly increased transformation efficiency. Interestingly, this phenomenon was not observed when the plasmid encoding bb_h09 (lp28–3) was lost. They hypothesised that the shuttle vector used for transformation does not possess the recognition/cleavage site of bb_h09, or the enzymes expressed by this gene were not expressed or active in these clones (Rego et al., 2011).

In our previous study, we identified and characterised the enzymatic activity of all four RMS genes of *B. afzelii* and *B. garinii* (Ruivo et al., 2024). We also demonstrated that in vitro methylation of a vector before transformation in the *Bbss* B31 lab strain can significantly increase transformation efficiency without the need for a B31 strain lacking the native *Borrelia* RMS. This led to our hypothesis that pre-methylation might also increase transformation efficiency in early passage isolates, which are closer to naturally occurring strains, forming the basis of the current study.

To this end, we focused on analysing and comparing all available data of Borrelia RMS genes in the REBASE database. In total, we analysed 18 *Borrelia* species, 7 species associated with LB, 10 species associated with RF and one species belonging to the Echidna-reptile group (Table 1). To date, REBASE does not possess data on all *Borrelia* species, only on the 18 species used in this study. This could be a limitation for this study, as it does not allow us to conclude on the presence and characteristics of RMS genes of all currently identified *Borrelia* species.

Between these three groups of species, differences in the number and composition of RMS genes were observed. In general, LB species feature three or four genes, in comparison to RF and Echidna-reptile group species that have two genes. The location and characteristics of these genes differ between the groups. In LB, all genes encode for a type II RMS and are located on linear plasmids. On the other hand, in RF and Echidna-reptile group species, one gene located on the chromosome encodes for a methyltransferase without endonuclease activity, and the other located in a linear plasmid encodes for a type II RMS that conveys both methyltransferase and endonuclease activity. This difference proves the evolutionary deviation between the groups.

Genetic diversity between the three groups is likely to be the result of factors such as habitat, geographical region, host and vector species. Due to differential gene expression, *Borrelia* can adapt and survive in different host species, often representing fundamentally different environments (Król et al., 2022). Most RF *Borrelia* species are transmitted by soft ticks and have a limited number of vertebrate hosts (Philip E. Stewart et al., 2022). Also, these species have a more stable genome with no plasmid loss reported during prolonged culture passage (Gilmore et al., 2021). In contrast, LB species are associated with a large variety of different hard tick species and vertebrate hosts and have shown a high plasticity in the plasmid content (Król et al., 2022; Margos et al., 2017). We hypothesise that the higher number of RMS genes of LB species could be related, on the one hand, to their higher prevalence in different tick species and hosts and the necessity to adapt rapidly to these different environments. On the other hand, a higher number of RMS genes might reflect the high degree of heterogeneity and plasticity in terms of plasmid content observed in this *Borrelia* group (Becker et al., 2020). Since these species have the tendency to lose plasmids during propagation, a higher number of RMS genes all present in different plasmids would ensure that even if some plasmids are lost, the defence mechanism against phages is maintained by at least one gene. We observed in our previous studies that every tested *Borrelia* isolate had at least one RMS gene variant present. This way, we speculate the importance of this defence mechanism for the survival of Borrelia (Ruivo et al., 2024).

Two RF species are not transmitted by soft ticks: *B. recurrentis,* which is transmitted from human to human by body lice and *B. miyamotoi,* which is transmitted by hard ticks. Interestingly, the RMS of these two species is quite different from the other RF species. *Borrelia recurrentis* has only one gene present in the chromosome that encodes for a methyltransferase and no type II RMS. On the other hand, *B. miyamotoi* has one gene present in the linear plasmid that has a similarity of 93.4 % at the amino acid level with the conserved RMS gene of LB species. Even though *B. miyamotoi* is grouped phylogenetically with RF spirochetes; it has some similarities with the LB species, including the vectors and part of the RMS (Trevisan et al., 2021). The gene present in the linear plasmid of *B. miyamotoi* is phylogenetically closely related to the RMS genes of the LB group, whereas the gene present in the chromosome clusters together with all the methyltransferase genes encoded in the chromosome of the RF species (Fig. 1).

One of the clades in the constructed phylogenetic tree (Fig. 1) incorporates a highly conserved RMS gene that is present in the plasmid of all the LB species and *B. miyamotoi.* This clade has two major clusters, one that includes the species predominantly found in Europe, such as *B. afzelii, B. garinii, B. miyamotoi* and *B. spielmanii.* The other cluster includes species that are mostly found in the USA, such as *Bbss, B. mayonii* and *B. bissettii.* All the species mentioned can be found in rodents and similar species of ticks, which means that a genetic interchange can happen between the different species of *Borrelia* inside the host or vector. In a previous study by Mongodin *et al,* three phylogenetic trees were constructed for the *B. burgdorferi* sensu lato complex based on the chromosome, cp26 and lp54. In all three of these trees, they observed that the species within the same continent are phylogenetically more closely related (Mongodin et al., 2013). Similarly, the clusters within clade 1 in our phylogenetic tree were also geographically defined.

Previous studies reported a third group of *Borrelia, the* Echidna-Reptile group, that does not cluster phylogenetically with any of the other two groups (Trevisan et al., 2021). Likewise, in our study, we observed that the methyltransferase of *B. turcica* located on the main chromosome does not form a clade with any of the other RMS genes. On the other hand, the type II RMS of this species clusters together with a variety of different RMS genes, both from LB and RF (Fig. 1) groups. One of the clades (clade 5) incorporates all the methyltransferases encoded in the chromosomes of the RF species. This clade shows a higher phylogenetic relationship between the species present in Africa (*B. hispanica, B. crocidurae, B. duttonii,* and *B. recurrentis)* compared to the ones present in the USA and Europe (*B. parkeri, B. turicatae, B. miyamotoi* and *B. hermsii*). Phylogenetic data demonstrated that RF and the Echidna-Reptile group are genetically similar but distinct, forming independent clades that share a common ancestor. Previously, it was shown that the linear chromosome of *Borrelia* expresses essential genes, and it is exceptionally stable (Tyler et al., 2018; Villa & de Miguel Bouzas, 2021). In the current study, we show that the RMS genes present in the chromosome form clades depending on the *Borrelia* group. For genes located on the plasmids, higher genetic diversity in all three groups of *Borrelia* is observed, with no evident group clades being formed. Since the main chromosome is highly stable, we hypothesise that it is unlikely that recombination will occur within this region between species of the three groups. Thus, the RMS gene present in the chromosome, or its lack, together with biological characteristics, can be used to distinguish between the *Borrelia* groups.

Most of the previous transformation efficiency studies were performed using *Bbss* B31 (Casselli et al., 2018; Chen et al., 2008; Rego et al., 2011; Ruivo et al., 2024). This strain is considered a laboratory-adapted strain with fewer plasmids, and due to repeated in vitro passage, can lose the ability to infect and cause disease (S. J. Norris et al., 1992). In contrast, patient isolates usually are low-passage strains that are likely to have retained their original plasmid content due to limited cultivation and can contain several proteins that are absent or expressed in smaller quantities in high-passage strains, including the RMS (Steven J. Norris et al., 2011). This way, low-passage strains are a better genetic representation of the spirochetes that are found in the environment.

In this study, we implemented our previously described methods that allowed us to increase the transformation success rate of low-passage borrelial (patient) isolates (Ruivo et al., 2024). By in vitro methylation of the vector prior to transformation in *Borrelia*, we were able to substantially increase the transformation efficiency. For the *B. garinii* #1226 isolate, the transformation efficiency was not statistically significant (p=0.061), due to the smaller number of positive transformants. Our hypothesis is that the observed increase in transformation efficiency in all low-passage strains when using an in vitro methylated vector is associated with the vector being recognised by the RMS of the borrelial strain as “native-DNA” and, thus, is not degraded by the endonuclease. More research will be needed to evaluate if the number and/or characteristics of RMS genes present in individual strains influences the transformation efficiency, or if the difference that we observed is only due to specific strain characteristics.

## 5 Conclusion

Low transformation efficiency with low passage *Borrelia* strains was a limiting factor for genetic manipulation methods such as transposon mutagenesis, allelic exchange and Cas9-based approach or even an adapted clustered regularly interspaced palindromic repeats interference (CRISPRi) platform (Takacs et al., 2021, 2022). Therefore, in vitro methylation has the potential to be a widespread tool to ease the study of the biology and pathogenesis of *Borrelia*, as also suggested by other studies (Gilmore et al., 2021; Margos et al., 2017). The knowledge gained in this study allows us to genetically modify low-passage infectious borrelial (patient) isolates and enable further host-pathogen and tick interaction studies. To the best of our knowledge, our study shows for the first time a high transformation success of low passage *B. afzelii* and *B. garinii* isolates.

## CRediT authorship contribution statement

**Margarida Ruivo:** Data curation, Formal analysis, Investigation, Methodology, Validation, Visualization, Writing – original draft. **Anna-Margarita Schötta:** Formal analysis, Investigation, Writing – review & editing. **Theresa Stelzer:** Investigation, Writing – review & editing. **Michael Reiter:** Methodology, Writing – review & editing. **Michiel Wijnveld:** Conceptualization, Data curation, Funding acquisition, Methodology, Project administration, Resources, Supervision, Validation, Visualization, Writing – original draft, Writing – review & editing.

## Declaration of competing interest

The authors declare the following financial interests/personal relationships which may be considered as potential competing interests:

Michiel Wijnveld reports financial support was provided by Austrian Science Fund. Reports a relationship with that includes:. Has patent pending to. If there are other authors, they declare that they have no known competing financial interests or personal relationships that could have appeared to influence the work reported in this paper.

## Data Availability

The data supporting the findings of this study are available in the Article and Supplementary Information. The sequence of the genes mentioned in this study can be found through the accession numbers mentioned in Table 1 on the NCBI database.

## References

[bib0001] Becker N.S., Rollins R.E., Nosenko K., Paulus A., Martin S., Krebs S., Takano A., Sato K., Kovalev S.Y., Kawabata H., Fingerle V., Margos G. (2020). High conservation combined with high plasticity: genomics and evolution of Borrelia bavariensis. BMC Genom..

[bib0002] Casselli T., Tourand Y., Scheidegger A., Arnold W.K., Proulx A., Stevenson B., Brissette C.A. (2018). DNA methylation by restriction modification systems affects the global transcriptome profile in Borrelia burgdorferi. J. Bacteriol..

[bib0003] Chan K., Alter L., Barthold S.W., Parveen N. (2015). Disruption of bbe02 by insertion of a luciferase gene increases transformation efficiency of Borrelia burgdorferi and allows live imaging in lyme disease susceptible C3H mice. PLoS. One.

[bib0004] Chen C., Zhang Y., Chen R., Liu K., Wu H., Qiao J., Caiyin Q. (2025). Development of a pre-modification strategy to overcome restriction–Modification barriers and enhance genetic engineering in lactococcus lactis for nisin biosynthesis. Int. J. Mol. Sci..

[bib0005] Chen Q., Fischer J.R., Benoit V.M., Dufour N.P., Youderian P., Leong J.M. (2008). In vitro CpG methylation increases the transformation efficiency of Borrelia burgdorferi strains harboring the endogenous linear plasmid lp56. J. Bacteriol..

[bib0006] Chiappa G., Perini M., Cafiso A., Nodari R., Wilhelmsson P., Lindgren P.-E., Omazic A., Ullman K., Moutailler S., Kjellander P., Bazzocchi C., Grandi G. (2022). A novel high discriminatory protocol for the detection of Borrelia afzelii, Borrelia burgdorferi Sensu Stricto and Borrelia garinii in ticks. Pathogens..

[bib0007] Faith D.R., Kinnersley M., Brooks D.M., Drecktrah D., Hall L.S., Luo E., Santiago-Frangos A., Wachter J., Samuels D.S., Secor P.R. (2024). Characterization and genomic analysis of the Lyme disease spirochete bacteriophage ϕbb-1. PLoS. Pathog..

[bib0008] Finn M.B., Ramsey K.M., Tolliver H.J., Dove S.L., Wessels M.R. (2021). Improved transformation efficiency of group A Streptococcus by inactivation of a type I restriction modification system. PLoS. One.

[bib0009] Gilmore R.D., Mikula S., Harris E.K., Van Gundy T.J., Goodrich I., Brandt K.S. (2021). Borrelia miyamotoi strain LB-2001 retains plasmids and infectious phenotype throughout continuous culture passages as evaluated by multiplex PCR. Ticks. Tick. Borne Dis..

[bib0010] Huang, Y., Wang, X., Chen, M., Wu, Y., Todhanakasem, T., Peng, N., He, M., & Wu, B. (2025). Bioresource Technology Engineering Zymomonas mobilis for improving genetic transformation and stability of multi-gene biosynthetic pathways. 438(July). 10.1016/j.biortech.2025.133198 40850582

[bib0011] James A.E., Rogovskyy A.S., Crowley M.A., Bankhead T. (2016). Characterization of a DNA adenine methyltransferase gene of Borrelia hermsii and its dispensability for murine infection and persistence. PLoS. One.

[bib0012] Kawabata H., Norris S.J., Watanabe H. (2004). BBE02 disruption mutants of Borrelia burgdorferi B31 have a highly transformable, infectious phenotype. Infect. Immun..

[bib0013] Koutantou M., Drancourt M., Angelakis E. (2024). Prevalence of Lyme disease and relapsing fever borrelia spp. in vectors, animals, and humans within a one health approach in Mediterranean countries. Pathogens.

[bib0014] Król N., Obiegala A., Imholt C., Arz C., Schmidt E., Jeske K., Ulrich R.G., Rentería‑Solís Z., Jacob J., Pfeffer M. (2022). Diversity of Borrelia burgdorferi sensu lato in ticks and small mammals from different habitats. Parasit. Vectors.

[bib0015] Kurokawa C., Lynn G.E., Pedra J.H.F., Pal U., Narasimhan S., Fikrig E. (2020). Interactions between Borrelia burgdorferi and ticks. Nat. Rev. Microbiol..

[bib0016] Lawrenz M.B., Kawabata H., Purser J.E., Norris S.J. (2002). Decreased electroporation efficiency in Borrelia burgdorferi containing linear plasmids lp25 and lp56: impact on transformation of infectious B. burgdorferi. Infect. Immun..

[bib0017] Lopez J., Hovius J.W., Bergström S. (2021). Pathogenesis of relapsing fever. Curr. Issues. Mol. Biol..

[bib0018] Madhusoodanan U.K., Rao D.N. (2010). Diversity of DNA methyltransferases that recognize asymmetric target sequences. Crit. Rev. Biochem. Mol. Biol..

[bib0019] Margos G., Hepner S., Mang C., Marosevic D., Reynolds S.E., Krebs S., Sing A., Derdakova M., Reiter M.A., Fingerle V. (2017). Lost in plasmids: next generation sequencing and the complex genome of the tick-borne pathogen Borrelia burgdorferi. BMC Genom..

[bib0020] Margos Gabriele, Gofton A., Wibberg D., Dangel A., Marosevic D., Loh S.M., Oskam C., Fingerle V. (2018). The genus Borrelia reloaded. PLoS. One.

[bib0021] Marques A.R., Strle F., Wormser G.P. (2021). Comparison of lyme disease in the United States and Europe. Emerg. Infect. Dis..

[bib0022] Mongodin E.F., Casjens S.R., Bruno J.F., Xu Y., Drabek E.F., Riley D.R., Cantarel B.L., Pagan P.E., Hernandez Y.A., Vargas L.C., Dunn J.J., Schutzer S.E., Fraser C.M., Qiu W.G., Luft B.J. (2013). Inter- and intra-specific pan-genomes of Borrelia burgdorferi sensu lato: genome stability and adaptive radiation. BMC Genom..

[bib0023] Norris S.J., Carter C.J., Howell J.K., Barbour A.G. (1992). Low-passage-associated proteins of Borrelia burgdorferi B31: characterization and molecular cloning of OspD, a surface-exposed, plasmid- encoded lipoprotein. Infect. Immun..

[bib0024] Norris Steven J., Howell J.K., Odeh E.A., Lin T., Gao L., Edmondson D.G. (2011). High-throughput plasmid content analysis of Borrelia burgdorferi B31 by using luminex multiplex technology. Appl. Env. Microbiol..

[bib0025] Rego R.O.M., Bestor A., Rosa P.A. (2011). Defining the plasmid-borne restriction-modification systems of the lyme disease spirochete Borrelia burgdorferi. J. Bacteriol..

[bib0026] Reiter M., Schötta A.M., Müller A., Stockinger H., Stanek G. (2015). A newly established real-time PCR for detection of Borrelia miyamotoi in Ixodes ricinus ticks. Ticks. Tick. Borne Dis..

[bib0027] Rosa P.A., Jewett M.W. (2020). Genetic manipulation of Borrelia. Curr. Issues. Mol. Biol..

[bib0028] Ruivo M., Kovács N.Z., Schötta A., Stelzer T., Hermann L., Mündler V., Bergthaler A., Reiter M., Wijnveld M. (2024). Optimising transformation efficiency in Borrelia: unravelling the role of the restriction-Modification system of Borrelia afzelii and Borrelia garinii. Int. J. Mol. Sci..

[bib0029] Samuels D.S. (1995). Electrotransformation of the spirochete Borrelia burgdorferi. Methods Mol. Biol..

[bib0030] Schwartz I., Margos G., Casjens S.R., Qiu W.G., Eggers C.H. (2021). Multipartite genome of lyme disease borrelia: structure, variation and prophages. Curr. Issues. Mol. Biol..

[bib0031] Shan J., Jia Y., Teulières L., Patel F., Clokie M.R.J. (2021). Targeting multicopy prophage genes for the increased detection of Borrelia burgdorferi Sensu Lato (s.l.), the causative agents of Lyme disease, in blood. Front. Microbiol..

[bib0032] Shon Y.J., Baek D., Jin S.Bin, Kim W.J., Jung G.Y., Lim H.G (2025). Development of a CRISPR-based cytosine base editor for restriction-modification system inactivation to enhance transformation efficiency in Vibrio Sp. dhg. J. Biol. Eng..

[bib0033] Stanek G., Wormser G.P., Gray J., Strle F. (2012). Lyme borreliosis. Lancet.

[bib0034] Stewart P.E., Thalken R., Bono J.L., Rosa P. (2001). Isolation of a circular plasmid region sufficient for autonomous replication and transformation of infectious Borrelia burgdorferi. Mol. Microbiol..

[bib0035] Stewart Philip E., Raffel S.J., Gherardini F.C., Bloom M.E. (2022). Kinetics of tick infection by the relapsing fever spirochete Borrelia hermsii acquired through artificial membrane feeding chambers. Sci. Rep..

[bib0036] Takacs C.N., Nakajima Y., Haber J.E., Jacobs-Wagner C. (2022). Cas9-mediated endogenous plasmid loss in Borrelia burgdorferi. PLoS. One.

[bib0037] Takacs C.N., Scott M., Chang Y., Kloos Z.A., Irnov I., Rosa P.A., Liu J., Jacobs-Wagner C. (2021). A CRISPR interference platform for selective downregulation of gene expression in Borrelia burgdorferi. Appl. Env. Microbiol..

[bib0038] Trevisan G., Cinco M., Trevisini S., Di Meo N., Chersi K., Ruscio M., Forgione P., Bonin S. (2021). Borreliae part 1: borrelia lyme group and echidna-reptile group. Biology.

[bib0039] Tyler S., Tyson S., Dibernardo A., Drebot M., Feil E.J., Graham M., Knox N.C., Lindsay L.R., Margos G., Mechai S., Van Domselaar G., Thorpe H.A., Ogden N.H. (2018). Whole genome sequencing and phylogenetic analysis of strains of the agent of Lyme disease Borrelia burgdorferi from Canadian emergence zones. Sci. Rep..

[bib0040] Vasu K., Rao D.N., Nagaraja V. (2019).

[bib0041] Villa, T.G., & de Miguel Bouzas, T. (2021). Developmental biology in prokaryotes and lower eukaryotes. In Developmental Biology in Prokaryotes and Lower Eukaryotes. 10.1007/978-3-030-77595-7.

